# Using the EQ-5D-5L to investigate quality-of-life impacts of disease-modifying therapy policies for people with multiple sclerosis (MS) in New Zealand

**DOI:** 10.1007/s10198-022-01518-x

**Published:** 2022-09-23

**Authors:** Suzi Claflin, Julie A. Campbell, Richard Norman, Deborah F. Mason, Tomas Kalincik, Steve Simpson-Yap, Helmut Butzkueven, William M. Carroll, Andrew J. Palmer, C. Leigh Blizzard, Ingrid van der Mei, Glen J. Henson, Bruce V. Taylor

**Affiliations:** 1 Menzies Institute for Medical Research, University of Tasmania, Medical Science Precinct, 17 Liverpool Street, Hobart, TAS 7000 Australia; 2 Curtin University, Perth, Australia; 3 New Zealand Brain Research Institute, Christchurch, New Zealand; 4 CORe The University of Melbourne, Melbourne, Australia; 5 Department of Neurology, The Royal Melbourne Hospital, Melbourne, Australia; 6 Neuroepidemiology Unit, Melbourne School of Population and Global Health, The University of Melbourne, Melbourne, Australia; 7 Department of Neuroscience, Monash University, Melbourne, Australia; 8 Perron Institute, Nedlands, Australia; 9 Centre for Health Policy, School of Population and Global Health, The University of Melbourne, Melbourne, Australia

**Keywords:** Multi-attribute utility instrument, Quality of life, Health technology assessment, Cost-utility analysis, Cost-effectiveness analysis, I110

## Abstract

**Background:**

Health state utilities (HSU) are a health-related quality-of-life (HRQoL) input for cost-utility analyses used for resource allocation decisions, including medication reimbursement. New Zealand (NZ) guidelines recommend the EQ-5D instruments; however, the EQ-5D-5L may not sufficiently capture psychosocial health. We evaluated HRQoL among people with multiple sclerosis (MS) in NZ using the EQ-5D-5L and assessed the instrument’s discriminatory sensitivity for a NZ MS cohort.

**Methods:**

Participants were recruited from the NZ MS Prevalence Study. Participants self-completed a 45-min online survey that included the EQ-5D-5L/EQ-VAS. Disability severity was classified using the Expanded Disability Status Scale (EDSS) to categorise participant disability as mild (EDSS: 0–3.5), moderate (EDSS: 4.0–6.0) and severe (EDSS: 6.5–9.5). Anxiety/depression were also measured using the Hospital Anxiety and Depression Score (HADS). In the absence of an EQ-5D-5L NZ tariff, HSUs were derived using an Australian tariff. We evaluated associations between HSUs and participant characteristics with linear regression models.

**Results:**

254 participants entered the study. Mean age was 55.2 years, 79.5% were female. Mean (SD) EQ-5D-5L HSU was 0.58 (0.33). Mean (SD) HSUs for disability categories were: mild 0.80 ± 0.17, moderate 0.57 ± 0.21 and severe 0.14 ± 0.32. Twelve percent reported HSU = 1.0 (i.e., no problems in any domain). Participants who had never used a disease-modifying therapy reported a lower mean HSU. Multivariable modelling found that the HADS anxiety score was not associated with EQ-5D-5L.

**Conclusions:**

HRQoL for people with MS in NZ was lower than comparable countries, including Australia. We suggest a comparison with other generic tools that may have improved sensitivity to mental health.

## Introduction

Multiple sclerosis (MS) is an inflammatory, neurodegenerative disease of the central nervous system that leads to increasing disability over time and reduced health-related quality of life (HRQoL) [1]. MS onset typically occurs in early adulthood, negatively impacting lifestyle and function across the life course [2]. Consequently, the impact of MS on health-related HRQoL can be acute and long-lasting on outcomes such as independent living and employment outcomes for both the individual and society [3]. Broader societal impacts include negative productivity effects on both paid and unpaid employment, and reduced community engagement [4].

The prevalence of MS is increasing worldwide (2.8 million people) with [5]. Increased prevalence can be attributed to a number of factors, including increased incidence, case longevity and increased detection of MS [6]. MS prevalence in New Zealand (NZ) is increasing; studies spanning 40 years have found that the prevalence of MS within the same regions of NZ has significantly increased while the sex ratio and latitudinal gradient have remained stable [7, 8].

Health state utilities (HSUs) are used to reflect HRQoL and as an input metric to cost-utility analysis (CUA) for resource allocation decisions [9]. Utilities have also been shown to be independent predictors of patient outcomes, including all-cause mortality and development of complications [10]. Moreover, clinicians have found that measuring HSUs is of benefit to patients regarding clinical assessment, relationships, communication, and management [11]. HSUs are values that measure the strength of preference for a particular health state, represented as a number between 0 and 1 where 0 is anchored to death (or health states equivalent to being dead) and 1 corresponds to perfect health. Notably, health states worse than death are possible, with negative utilities assigned [12].

Resourcing decisions, particularly for disease-modifying therapies (DMTs, a medication class used to treat MS) for MS are typically based on CUA for Health Technology Assessments (HTA), which utilise HSUs. Several multi-attribute utility instruments (MAUIs) are available from which utilities can be derived for use in CUA, including the EQ-5D-5L [13]. The EQ-5D-5L (and its predecessor, the EQ-5D-3L) is used in over 63% [14] of economic evaluations and recommended for CUA in over 85% of HTA guidelines worldwide [15].

### New Zealand and the EQ-5D-5L for reimbursement decisions

Official pharmacoeconomic guidelines inform manufacturers and others on which methods to follow regarding CUA to support applications for access, reimbursement, or pricing, including for MS-specific DMTs [15]. There is no international consensus about the content of pharmacoeconomic guidelines, so recommendations differ between countries [15].

New Zealand’s Pharmaceutical Management Agency (PHARMAC) guidelines “*Prescription for Pharmacoeconomic Analysis. Methods for Cost-Utility Analysis (Version 2.2)*” recommends the use of the EQ-5D suite of instruments for HTA and states that “The EQ-5D is widely used internationally, and utility weights have been derived from the New Zealand population” [15]. In contrast for example, the guidelines of the Australian Pharmaceutical Benefits Advisory Committee (PBAC), “*Guidelines for Preparing a Submission to the Pharmaceutical Benefits Advisory Committee (Version 5.0)*”, are less prescriptive and recommend the use of a suite of MAUIs where utility weights have been derived for the domestic population, including the EQ-5D (3L and 5L), HUI2 or HUI3, SF-6D, AQoL, and CHU9D [15].

### Use of the EQ-5D-5L in MS study populations

Overall, the EQ-5D-5L has been found to be sub-optimal in capturing complex psychosocial health status for people with complex and chronic disease [16]. With respect to MS study populations, the use of the EQ-5D-5L has been limited worldwide despite the instrument’s high prevalence in HTAs [15]. This is reflected in Australia, where there is limited EQ-5D-5L evidence in MS study populations. Our group previously mapped EQ-5D-5L utilities from the WHOQOL-100 and this derivation reported a mean HSU for Australians living with MS of 0.54 utility and those with a severe disability at a mean of 0.41 and those with mild disability at a mean score of 0.62 [17]. A more recent study by our group adopted the EQ-5D-5L and this study established a mean HSU for those with progressive MS of 0.54 and a mean HSU for those with relapsing–remitting MS of 0.73 [18]. In NZ, the use of the EQ-5D-5L for pwMS has not been reported in the scholarly literature.

## Materials and methods

### The CompANZ study and our study aims

The Comparing MS Populations in Australia and New Zealand (CompANZ) study is an observational cohort study that collected data from two extant cohorts, one in each nation and the study cohort was recruited as part of the CompANZ study that has been described in detail elsewhere [19]. In brief, the NZ cohort of the CompANZ study was recruited as a ten-year follow-up to the NZ MS Prevalence Study (NZMSPS). The primary aim of the NZMSPS was to determine the prevalence and distribution of pwMS in NZ on census day 7 March 2006 and has been reported extensively elsewhere including ethics approvals [7].

The inclusion criteria for the CompANZ study were: (a) previous participation in the NZMSPS; (b) relapsing-onset MS; and (c) MS diagnosis between 1 January 1996 and 31 December 2006. The main aim of thestudy was to evaluate (1) the impact of national-level DMT subsidy policy on DMT use and health outcomes among people living with MS (pwMS); and (2) to evaluate long-term effects of DMT use on health outcomes among pwMS. The study was conducted from 2017 to 2018.

The aims of our current study are twofold. First, we explored HRQoL among the NZ CompANZ cohort using the EQ-5D-5L. Second, we investigated the discriminatory sensitivity of the EQ-5D-5L among pwMS in NZ.

### Study recruitment

As described elsewhere [19], we followed-up NZMSPS participants who met the inclusion criteria, and who had consented to participate in future research using contact details provided during the NZMSPS from March 2017 to February 2018. People with outdated contact details or whom we were unable to contact directly were also contacted using details associated with their National Health Index (NHI) number. All phone numbers and email addresses were tried at least once.

### Measurements

As described below, patient-reported outcome data, socio-demographic and clinical data were collected in a single questionnaire that took approximately 45 min to complete. Participants were given the choice of completing the survey over the phone or online.

#### Outcome measure: EQ-5D-5L health state utilities and item scores, and EQ-VAS scores

The EQ-5D-5L was developed to address the limited sensitivity (lack of descriptive richness and serious ceiling effects) of its predecessor the EQ-5D-3L [20] and describes 3,125 health states. The algorithmic range for most of the instrument’s country specific value sets describe HSUs ranging from < ‘0’ to ‘1.0’ [12]. The EQ-5D-5L also uses a visual analogue scale (EQ-VAS) in which participants rate their current health state on a scale of 0–100 (worst to best imaginable health) [16].

The primary outcome measure of this study was HRQoL captured by and assessed with the EQ-5D-5L’s HSU and individual item scores regarding mobility, self-care, usual activities, pain, and anxiety/depression (1 (best response) to 5 (worst response)), and the EQ-VAS scores (scale 0–100) [21].

In the absence of a EuroQoL-approved NZ country-specific value set [22, 23], we derived HSUs for our study population using the most recently reported Australian value set [24]. Population norms that accord with the Australian value set were sourced from the literature with the mean (SD) Australian population norm reported as 0.91 (0.14) utility points [25]. We also established from the literature that the EQ-5D-3L NZ population norm for the general population is 0.85 utility points [23, 26] and for the EQ-VAS is 74.8 points [23]. We adopted a conservative estimate of the minimal important difference (MID) for the EQ-5D-5L as 0.04 utility points [27]. The estimate is conservative because we adopted the lower bounds of the country specific MIDs [27].

#### Other patient-reported health outcome measures

The survey also assessed disability, fatigue, anxiety, and depression. Disability was measured using the Expanded Disability Status Scale (EDSS) and the Multiple Sclerosis Severity Score (MSSS). Both instruments primarily assess mobility and physical health, not psychosocial health. The survey also queried year of MS diagnosis to determine disease duration and measured current EDSS via the web-EDSS [28] (a validated online version of the tool), and thus calculated the MSSS, a relative measure of disease progression [29]. Both tools are 0–10 scales, where 10 indicates death. In this study, we categorised participant disability as mild (EDSS 0–3.5), moderate (EDSS 4.0–6.0; EDSS of 6.0 indicates that the participant requires assistance to walk), or severe disability (EDSS 6.5–9.5) [17]. Fatigue was measured using the Fatigue Severity Scale (FSS) [30], and anxiety and depression were measured using the Hospital Anxiety and Depression Score (HADS) [31]. The FSS is a 9-item scale; mean scores range from 1 to 7, with scores > 5 indicating clinically significant fatigue. The HADS is a 14-item scale, with anxiety and depression scores ranging from 0 to 21. Scores may be categorised as normal (0–7), borderline abnormal (8–10), or abnormal (11–21).

#### Disease-modifying therapy variables

DMT use was queried; participants were presented with a list of DMTs and reported the total number of months of use for each one and the self-reported time between diagnosis and first DMT use. From this information, we derived four DMT variables: *time between diagnosis and first DMT use*, *total duration of DMT use*, *ever used DMT*, and *DMT treatment fraction*. *Ever used DMT* was a binary variable where all participants who reported ever using a DMT for ≥ 1 month were categorised as a DMT user. *DMT treatment fraction* was a measure of relative DMT use and was defined as the number of months of DMT treatment divided by the number of months of MS duration (calculated from year of diagnosis).

Implausible DMT exposure values (i.e., those that exceeded the amount of time that DMT had been available) were excluded from analyses of total duration of DMT use and DMT treatment fraction, but these participants were still categorised as DMT users for the *ever used DMT* variable.

#### Other measures

We also collected data on age, sex, relationship status, education level, employment status, household income, self-reported weight and height, physical activity (International Physical Activity Questionnaire-Short Form) [32], smoking status and number of cigarettes smoked, and vitamin D supplement use. Body mass index (BMI) was calculated from self-reported height and weight using the formula, weight (kg)/height (m)^2^.

### Analysis

#### Representativeness of the study cohort

The representativeness of the study cohort is reported in-depth elsewhere [19]. In brief, representativeness was determined by comparing the characteristics of (1) NZMSPS participants who met our inclusion criteria and could be contacted (invited) with those who were not contactable (not invited); and (2) invited people that completed the survey (completers) with those who did not (non-completers). Comparisons were made using standardised differences.

#### Participant characteristics and outcome variables

Participant characteristics and outcome variables, including utility value and item scores, are reported as frequencies and percentages, and means and standard deviations.

#### Associations between participant characteristics and utility values

We evaluate associations between utility values and participant characteristics (including DMT use and health outcome variables) using linear regression models. Because outcomes were markedly skewed, these were log-transformed to reduce heteroscedasticity; we used Box–Cox regression to identify transformation theta coefficients. All coefficients were back-transformed at the mean of model covariables.

We first evaluated the associations in univariable models. We then assessed the associations between predictor variables using Chi-square tests. Based on the results of those tests and the results of the univariable analyses, we developed a final multivariable model that included variables that were significantly associated (*p* < 0.05, 95% CI that do not cross zero) with utility values in univariable models, but excluding variables that were significantly associated with one another to reduce collinearity. We excluded variables with the greatest collinearity (i.e., were associated with the greatest number of other variables).

We assessed the association between participant utility values and the EQ-VAS using Spearman correlation.

All analyses were carried out in Stata/SE 16 (StataCorp, College Station, TX, USA).

## Results

### Flow of participants into the study

**Fig. 1:**
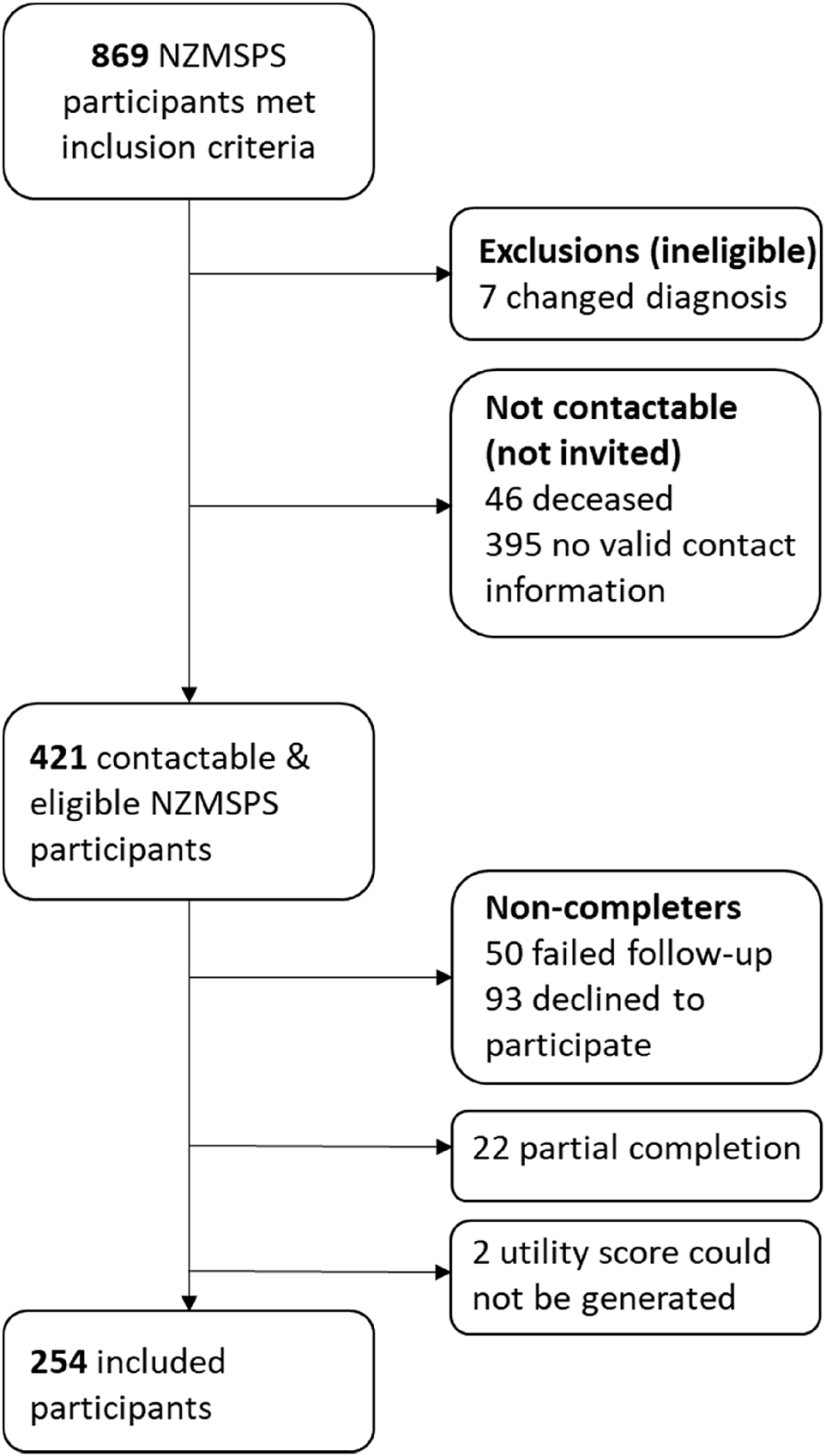
Inclusion flowchart, adapted from Claflin et al. [19]. Participants recruited as part of follow-up to the New Zealand MS Prevalence Study (NZMSPS)

Of the 869 NZMSPS participants who met the CompANZ study inclusion criteria, 421 (48.4%) were contactable and therefore invited to take part (Fig. 1). Of these, 254 (60.3%) completed the survey and provided enough information that an EQ-5D-5L utility value could be generated using the Australian value set. Previous analysis determined that those invited were reasonably representative of eligible NZMSPS participants, and that survey completers were reasonably representative of those invited [19].

### Participant characteristics

Participant characteristics are presented in Table 1. Most participants were middle-aged, female (79.5%), and partnered (72.4%). With respect to markers of socioeconomic status, more than two-thirds of the participants had less than a Bachelor’s degree (69.7%), about half were employed in paid work, and about half had a household income of ≥ NZ$1,000 per week.

**Table 1:** Characteristics of participants for whom a utility value could be generated (*N* = 254 unless otherwise noted)

Characteristic	*N* (%)
Sex	
Female	202 (79.5)
Male	52 (20.5)
Relationship status	
Partnered	184 (72.4)
Unpartnered	70 (27.6)
Vitamin D supplementation	
No	169 (66.5)
Yes	85 (33.5)
Smoking status	
No	231 (90.9)
Yes	23 (9.1)
Education level, *N* = 253	
Secondary school or less	94 (37.0)
Occupational diploma*	83 (32.7)
Bachelor's degree or greater	76 (29.9)
Missing	1 (0.4)
Employment status, *N* = 250	
Employed (paid work)	131 (51.6)
Unpaid work only	36 (14.2)
Retired	53 (20.9)
Unemployed	30 (11.8)
Missing	4 (1.6)
Household income (NZ$), *N* = *250*	
$0–299 per week	27 (10.6)
$300–599 per week	42 (16.5)
$600–999 per week	45 (17.7)
$1000–1999 per week	65 (25.6)
≥ $2000 per week	71 (28.0)
Missing	4 (1.6)
DMT treatment ever (binary)	
No	127 (50.0)
Yes	127 (50.0)
Disability categories	
Mild disability (0–3.5)	113 (44.5)
Moderate disability (4–6)	89 (35.0)
Severe disability (6.5–9.5)	52 (20.5)
Characteristic	Mean (SD)
Age at survey start	55.2 (9.7)
Disease duration (years), *N* = *239*	15.9 (3.4)
Body mass index (BMI), *N* = *251*	27.5 (10.4)
Fatigue and mental health	
Mean FSS	4.7 (1.7)
HADS anxiety score	6.3 (3.7)
HADS depression score	5.1 (3.5)
DMT outcomes	
DMT duration (months), *N* = *251*	41.0 (59.0)
Time from diagnosis to first DMT (months), *N* = *126*	54.6 (62.4)
Treatment fraction**, *N* = *247*	0.23 (0.33)
Disability outcomes	
MSSS, *N* = *239*	4.2 (2.6)
EDSS	4.2 (2.0)

*HADS*: Hospital Anxiety and Depression Scale, *DMT* disease modifying therapy, *EDSS* Expanded Disability Status Scale, *MSSS* Multiple Sclerosis Severity Score

*Occupational diploma: occupational or national certificate or diploma or associate degree

**Treatment fraction = number of months of DMT treatment/number of months disease duration (from diagnosis)

Most participants (79.5%) were living with mild to moderate disability, with a mean (SD) EDSS of 4.2 ± 2.0. Similarly, on average, participants were living with moderate fatigue, and normal levels of anxiety and depression (≤ 7 out of a possible 21 for the HADS). Half of the participants had ever taken a DMT, with the mean treatment fraction of 0.23, meaning that on average, participants were treated with a DMT a little less than a quarter of the time since their diagnosis. Further, on average, participants reported initiating DMT treatment more than 54 months (4.5 years) after diagnosis.

### EQ-5D-5L HSUs and item scores overall and by disease severity category, and EQ-VAS scores

**Fig. 2:**
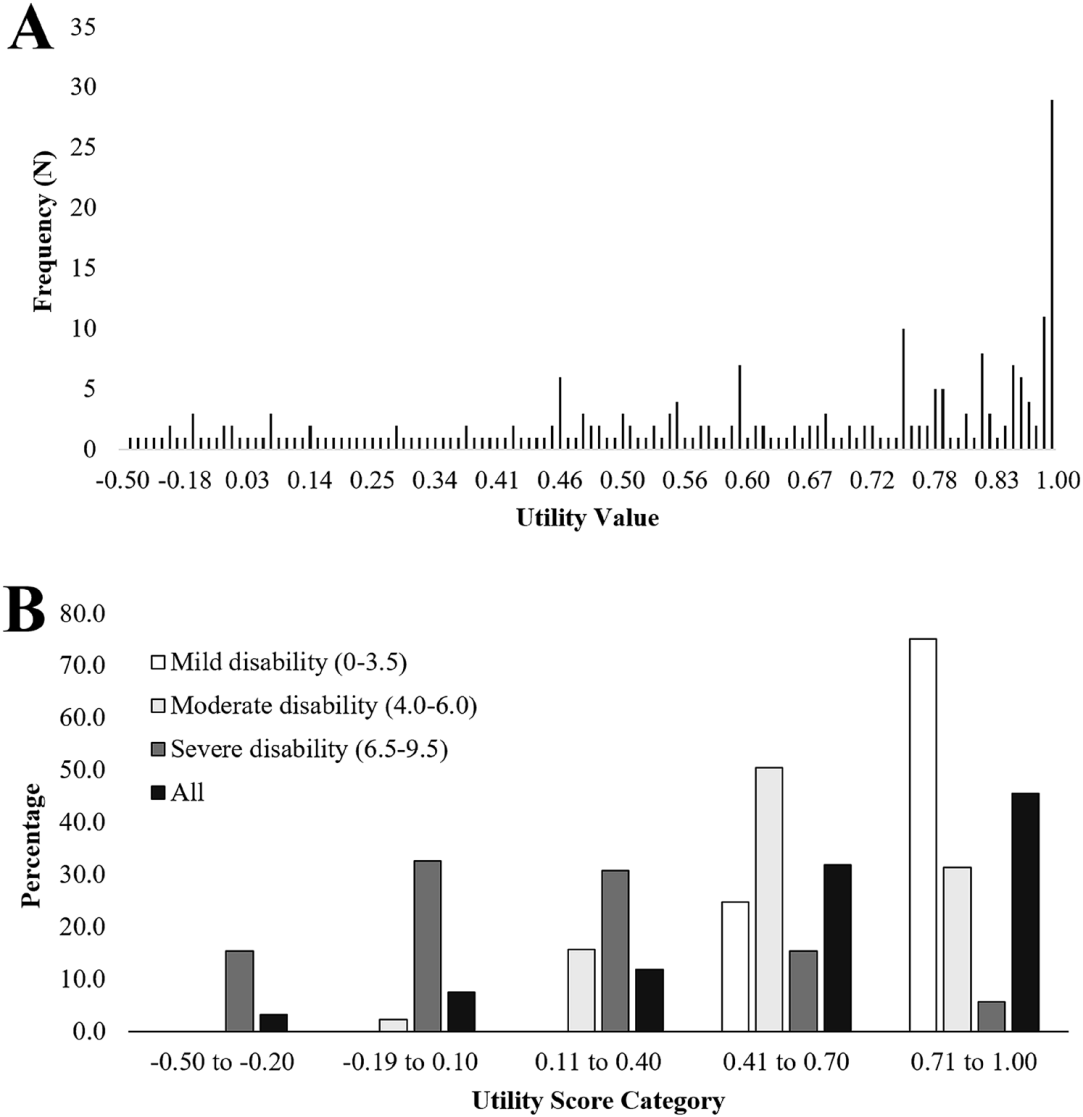
Histograms depicting **A** the frequency of utility values in the study cohort (*N* = 254); **B** the percentage of participants with utility values in given categories in the total cohort (*N* = 254); and the percentage of participants within a given disability category (mild: *n* = 113; moderate: *n* = 89; severe: *n* = 52) with a utility value within a given category

The mean EQ-5D-5L utility value for the entire cohort was 0.58 (SD 0.33; range: − 0.45, 1.00). The distribution of individual utility values was skewed negatively in this population (Fig. 2A); nearly half of the participants had a utility value > 0.70 (Fig. 2B). The mean (SD) EQ-VAS score (*N* = 252) was 69.4 (21.7) and was aligned with the EQ-5D-5L utility (Spearman’s rho: 0.59; *p* < 0.001).

Mean HSUs for the three disability categories were 0.80 (SD 0.17; range 0.41–1.00), 0.57 (SD 0.21; range 0.04–1.00) and 0.14 (SD 0.32; range: − 0.45 to 0.84), for mild, moderate, and severe disability, respectively. Differences between disability categories exceeded the MID for the EQ-5D-5L [27]. The distribution of utility values was also skewed among participants in different disability categories (Fig. 2B). Among participants with severe disability (*N* = 52), 79% had utility values ≤ 0.40. Further, all participants with utility values ≤ − 0.20 were living with severe disability. Conversely, about half (51%) of those living with moderate disability had utility values between 0.41 and 0.70 (the second highest category). The utility values of participants living with mild disability were skewed to the right, with 75% having utility values of ≥ 0.71.

With respect to ceiling effects, those participants who reported an HSU of 1.0 (perfect health, *N* = 29), twenty-eight of these people recorded a mean EQ-VAS score of 91 (SD 7.7; range 75–100). Interestingly, two of these twenty-nine ceiling effect participants were classified with moderate disability and their mean EQ-VAS score was 77.5 (SD 3.5; range 75–80).

**Fig. 3:**
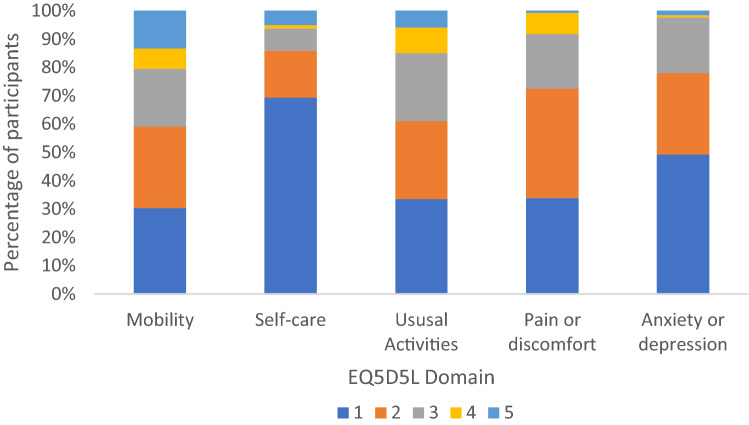
Percentage of participants who self-reported given item scores for the five EQ5D5L items in the study cohort (*N* = 254), where 1 is low symptom severity and 5 is high symptom severity

The frequency of reported severity for the five EQ-5D-5L items is presented in Fig. 3. Participants most commonly reported low to moderate severity across all items. Self-care was the item that participants were most likely to report as low severity (69%), followed by anxiety or depression (49%). Mobility was the item that participants were the most likely to report as high severity (13%).

### Univariable models of EQ-5D-5L HSUs

Univariable models show an association between utility values and employment status, household income, DMT use ever, disability category, and measures of fatigue and mental health (Table 2). Participants in unpaid employment statuses (unpaid work only, retired, unemployed) had significantly lower mean utility values than those in paid employment (full-time, part-time, or self-employed). Unemployed participants had a substantially lower mean HSU than participants in paid employment (0.27 compared to 0.73). Similarly, participants in the lowest income bracket (≤ NZ$299 per week) had a mean utility value that was about 1.4 times lower than the mean utility value of the top two income brackets (NZ$1000–1999 and ≥ NZ$2000 per week; 0.46 compared to 0.64 and 0.63, respectively). Participants who had ever used a DMT had 1.1 times lower mean utility value compared to those who had never been treated with a DMT (0.54 compared to 0.63) (Table 2).

**Table 2:** Results of linear regression models evaluating variables associated with EQ-5D-5L utility value in univariable models and a final multivariable model

			Univariable model		Multivariable model	
Characteristic	*N* (%)	Mean (SD; range)	*β* (95%CI)	*p *value	*β* (95%CI)	*p *value
All		0.58 (0.33; − 0.45, 1.00)				
Sex						
Female	202 (79.5)	0.58 (0.34; − 0.45, 1.00)	0.00 [Reference]			
Male	52 (20.5)	0.59 (0.31; − 0.27, 1.00)	− 0.001 (− 0.090, 0.089)	0.991		
Age Group						
36–47 years	65 (25.6)	0.62 (0.29; − 0.23, 1.00)	0.00 [Reference]			
48–55 years	64 (25.2)	0.62 (0.30; − 0.16, 1.00)	0.002 (− 0.098, 0.102)	0.973		
56–62 years	68 (26.8)	0.56 (0.36; − 0.45, 1.00)	− 0.050 (− 0.149, 0.050)	0.328		
63–80 years	57 (22.4)	0.53 (0.36; − 0.27, 1.00)	− 0.083 (− 0.189, 0.022)	0.121		
Test for trend				0.77		
Relationship status						
Partnered	184 (72.4)	0.60 (0.33; − 0.45, 1.00)	0.00 [Reference]			
Unpartnered	70 (27.6)	0.53 (0.32; − 0.29, 1.00)	0.0637 (− 0.157, 0.007)	0.75		
Education level, N = 253						
Secondary school or less	94 (37.0)	0.56 (0.36; − 0.27, 1.00)	0.00 [Reference]			
Occupational diploma*	83 (32.7)	0.58 (0.31; − 0.29, 1.00)	0.010 (− 0.078, 0.098)	0.826		
Bachelor's degree or greater	76 (29.9)	0.61 (0.32; − 0.45, 1.00)	0.041 (− 0.048, 0.130)	0.367		
Test for trend				0.372		
Employment status, N = 250						
Employed (paid work)	131 (51.6)	0.73 (0.25; − 0.45, 1.00)	0.00 [Reference]			
Unpaid work only	36 (14.2)	0.50 (0.27; − 0.18, 1.00)	**− 0.226 (− 0.324, − 0.129)**	** < 0.001**		
Retired	53 (20.9)	0.48 (0.34; − 0.29, 1.00)	**− 0.232 (− 0.315, − 0.148)**	** < 0.001**		
Unemployed	30 (11.8)	0.27 (0.36; − 0.28, 1.00)	**− 0.420 (− 0.537, − 0.301)**	** < 0.001**		
Test for trend				** < 0.001**		
Household income (NZ$), N = 250						
$0–299 per week	27 (10.6)	0.46 (0.40; − 0.29, 1.00)	0.00 [Reference]		0.00 [Reference]	
$300–599 per week	42 (16.5)	0.53 (0.31; − 0.27, 1.00)	0.057 (− 0.090, 0.204)	0.448	0.010 (− 0.065, 0.085)	0.802
$600–999 per week	45 (17.7)	0.56 (0.28; − 0.23, 1.00)	0.073 (− 0.071, 0.218)	0.321	0.027 (− 0.047, 0.101)	0.475
$1000–1999 per week	65 (25.6)	0.64 (0.34, − 0.45, 1.00)	**0.163 (0.028, 0.297)**	**0.018**	0.005 (− 0.066, 0.075)	0.899
≥ $2000 per week	71 (28.0)	0.63 (0.32; − 0.28, 1.00)	**0.154 (0.021, 0.287)**	**0.023**	0.010 (− 0.060, 0.079)	0.783
Test for trend				**0.003**		0.785
DMT treatment ever (binary)						
No	127 (50.0)	0.63 (0.32; − 0.27, 1.00)	0.00 [Reference]		0.00 [Reference]	
Yes	127 (50.0)	0.54 (0.34; − 0.45, 1.00)	**− 0.092 (− 0.164, − 0.021)**	**0.011**	**− 0.064 (− 0.104, − 0.024)**	**0.002**
Disability categories						
Mild disability (0–3.5)	113 (44.5)	0.80 (0.17; 0.41, 1.00)	0.00 [Reference]		0.00 [Reference]	
Moderate disability (4–6)	89 (35.0)	0.57 (0.21; 0.04, 1.00)	**− 0.226 (− 0.282, − 0.170)**	** < 0.001**	**− 0.117 (− 0.165, − 0.069)**	** < 0.001**
Severe disability (6.5–9.5)	52 (20.5)	0.14 (0.32; − 0.45, 0.839)	**− 0.627 (− 0.704, − 0.550)**	** < 0.001**	**− 0.498 (− 0.567, − 0.430)**	** < 0.001**
Test for trend				** < 0.001**		** < 0.001**
Fatigue and mental health						
Mean Fatigue Severity Scale score (1–7)			**− 0.091 (− 0.110, − 0.071)**	** < 0.001**	**− 0.043 (− 0.057, − 0.029)**	** < 0.001**
Hospital Anxiety and Depression Scale anxiety score (0–21)			**− 0.026 (− 0.036, − 0.017)**	** < 0.001**	− 0.005 (− 0.012, 0.001)	0.092
Hospital Anxeity and Depression Scale depression score (0–21)			**− 0.048 (− 0.057, − 0.039)**	** < 0.001**	**− 0.015 (− 0.023, − 0.008)**	** < 0.001**
DMT outcomes						
DMT duration (months), N = 251			− 0.000 (− 0.001, 0.000)	0.464		
Time from diagnosis to first DMT (months), N = 126			0.001 (− 0.000, 0.002)	0.097		
Treatment fraction**, N = 251			− 0.034 (− 0.146, 0.079)	0.556		

*P* values < 0.005 are bolded. *N* = 254, unless otherwise noted

*DMT* disease modifying therapy

*Occupational diploma: occupational or national certificate or diploma or associate degree

**Treatment fraction = number of months of DMT treatment/number of months disease duration (from diagnosis)

MS-related symptoms were also significantly associated with utility value. Participants with moderate or severe disability had significantly lower mean utility values than those with mild disability. Those with severe disability had a mean utility value that was almost six-times lower than those with mild disability (HSU of 0.14 compared to 0.80). Similarly, fatigue, depression, and anxiety scores were all negatively associated with utility value, meaning that participants with more severe symptoms had lower HRQoL, on average (Table 2).

However, utility value was not associated with age, sex, relationship status, education level, disease duration, or any continuous DMT outcome (total months on DMT, months between diagnosis and first DMT, and treatment fraction). However, as noted above, ever use of a DMT was associated with a higher HSU value (Table 2).

### Associations between predictor variables

We found that among variables that were significantly associated with utility values in univariable models, employment status was significantly associated with household income bracket (*χ*^2^ = 61.5; *p* < 0.001) and disability category (*χ*^2^ = 51.7; *p* < 0.001). However, household income bracket was not associated with disability category (*χ*^2^ = 12.0; *p* = 0.152). Further, disability category was not associated with DMT use ever (*χ*^2^ = 2.2; *p* = 0.341). Consequently, we excluded employment status from the final multivariable model, as it was collinear with both household income bracket and disability category. Instead, we included household income bracket as a measure of socioeconomic status in the final model.

### Multivariable model of health-related quality of life

Our final multivariable model yielded similar results to the univariable analyses. However, when the effect of disability category was accounted for, household income bracket was no longer significantly associated with utility value. Similarly, with the other variables accounted for, anxiety score was no longer significantly associated with utility value (Table 2).

Conversely, fatigue and depression scores remained significantly independently associated, resulting in decreases of 0.043 and 0.015 in utility value for every one unit increase in FSS or HADS score, respectively (Table 2).

## Discussion

To our knowledge, this is the first study to assess HRQoL for pwMS in NZ using a detailed analysis of EQ-5D-5L HSUs. HSUs are an input metric for quality-adjusted life years in CUA and are increasingly being used for patient management in value-based care and prediction of health status [10]. In the absence of an EQ-5D-5L country-specific value set (or population norms) for the NZ general population, we adopted a precedent from a clinical trial that incorporated a NZ cohort using the comparable Australian tariff [33]. Our study established that the mean HSU for pwMS in NZ (0.58 ± 0.33 utility points) was substantially less than EQ-5D-5L Australian population norms (0.91 ± 0.14 utility points [25]) and the NZ EQ-5D-5L population norms (0.85 utility points [23]). The difference between our study cohort mean and Australian and NZ population norms, exceeded the MID for the EQ-5D-5L, 0.04 utility points [27]. We found that people with severe MS in NZ reported a EQ-5D-5L utility value comparable with people with terminal cancer [34], or morbid and severe obesity [35]. We also found that as disability severity increased from mild to severe disability, the EQ-5D-5L HSU significantly decreased. Finally, we established that the mean EQ-VAS for the study population was 5.4 VAS points lower than the NZ population norm (69.4 compared to 74.8 points) [26].

### Comparison with other complex and chronic disease states in New Zealand and other MAUIs

There is a dearth of literature regarding the use of the EQ-5D-5L for people in NZ with complex and chronic disease. Furthermore, no NZ country-specific tariff exists for the EQ-5D-5L. Existing work has used the precursor to the 5L, the EQ-5D-3L and a 3L NZ-specific tariff [10, 36, 37]. A recent study (the ADRENAL clinical trial) used the EQ-5D-5L to investigate an international cohort of people (including NZ) for the impact of hydrocortisone treatment and illness severity on HRQoL six months after ICU admission for septic shock [33]. This study used the Australian tariff for each country [namely Australia, NZ (almost 12% of the trial cohort), Denmark, Saudi Arabia, and the United Kingdom] to report aggregated HSUs [33]. Importantly, the ADRENAL clinical trial supports the use of the Australian tariff for our study [33]. Notably, the results of this trial show that the mean HSU for the entire cohort of septic shock survivors was 0.59 utility points, which is comparable to our study’s findings regarding HSU for pwMS in NZ. Another recent study investigated SF-6D HSUs for a large international cohort of pwMS (called HOLISM: Health Outcomes and Lifestyle for a sample of pwMS) that included *N* = 168 participants from NZ. [1] This study found that the mean HSU for pwMS in NZ was 0.70 ± 0.12 utility points and that a number of lifestyle factors were associated with the SF-6D HSU including diet, physical activity, supplement use and smoking [1]. The difference between our estimates (0.12 utility points) may reflect differences in the study cohorts with a likely healthier participant bias in the HOLISM study cohort, but it may also reflect differences between MAUIs [38]. Notably, the absolute difference between the SF-6D and EQ-5D-5L is estimated to be 0.12 utility points [38].

### Discriminatory sensitivity of EQ-5D-5L for people with MS in New Zealand

We established an inverse relationship between EQ-5D-5L HSUs for pwMS in NZ and MS-related disability as measured by EDSS. This agrees with another study by our group, which mapped EQ-5D-3L HSUs for pwMS in Australia. In that study, HSU decreased with increasing disability: 0.61 (95% CI 0.60–0.62), 0.51 (95% CI 0.50–0.52) and 0.40 (95% CI 0.38–0.43) for mild, moderate, and severe disability, respectively [17]. This study also found that adjusted differences in mean HSU between the three severity groups were statistically significant [17]. The close association between the EQ-5D-5L may reflect the sensitivity of both instruments to physical health, particularly mobility, which was the domain most commonly rated as high severity in this cohort.

However, in contrast to other MAUIs that have been found to be preferentially sensitive to the complex psychosocial health needs of pwMS, our study’s multivariable modelling found that the anxiety score was no longer significantly associated with HSU when the model was adjusted for the effect of other factors. We also established that the ceiling effect for the EQ-5D-5L among pwMS in NZ is reduced from the ceiling effects reported in other study populations. However, 24 of the 28 people who generated a utility value of 1.0 (a MAUI that is weighted to physical health) rated themselves as having less than perfect health on the EQ-VAS, likely self-reporting a lower score by taking psychosocial health into account. We also found that 45% of the study population recorded a HSU of greater than 0.7 utility points.

Based on these preliminary findings using the Australian tariff for the EQ-5D-5L, we suggest that a NZ country-specific value set for the EQ-5D-5L be developed and validated for health technology assessment for pwMS in NZ. We also suggest that PHARMAC consider recommending other preferentially sensitive MAUIs for health technology assessments for pwMS in NZ.

### HTA policy in New Zealand may lead to poor reimbursement outcomes for people with MS

In addition to the comments made above regarding the discriminatory sensitivity of the EQ-5D-5L, we suggest that there is a real-world policy gap that may translate to poor policy outcomes regarding DMT prescription for pwMS in NZ. Namely, PHARMAC, the NZ reimbursement agency, states that the EQ-5D NZ tariff should be used to assess HSUs for CUA using the EQ-5D suite of MAUIs. However, a tariff does not exist for the most recent iteration of the EQ-5D, the EQ-5D-5L. Moreover, the existing tariff for the EQ-5D-3L is not an ideal instrument, as it has significant ceiling effects and is not preferentially sensitive to the HRQoL needs of people with complex and chronic disease [16, 39]. We suggest that this policy gap may lead to poor medication reimbursement outcomes for pwMS in NZ, and a concomitant diminished HRQoL as reflected in our study’s EQ-5D-5L HSUs.

### Lower HSU among people who used DMT likely results from indication bias

Our work suggests that among pwMS in NZ, those who had ever used a DMT had lower HRQoL than those who had never used one. This is likely an instance of indication bias, reflecting NZ DMT subsidy policy, which has restricted access to DMTs based on disability level and relapse rate [40]. Therefore, participants experiencing more severe disability and/or a more severe disease course were more likely to be eligible for DMT subsidies and consequently to use DMT. We suggest that future studies evaluate the impact of DMT use on HRQoL among recently diagnosed pwMS in NZ, who have had more permissive DMT subsidy policy early in their disease course.

### Employment status

In univariable models, we found that unemployed pwMS in New Zealand recorded a mean HSU that was substantially less than the mean HSU for people who were employed. Epidemiological work by our group using the Australian MS Longitudinal Study has established that work productivity is most strongly determined by symptoms, particularly ‘fatigue and cognitive symptoms’ and ‘pain and sensory symptoms’, while older age, and lower education level were also predictive of not being in the labour force [41]. We note that employment status was not included in our multivariable model and household income bracket was included. Nevertheless, when the effect of disability category was accounted for, household income bracket was no longer significantly associated with HSU and therefore HRQoL. We note that a large international study (including a NZ cohort) found that employment and higher socioeconomic status were significantly associated with higher HSU and therefore HRQoL [1]. We suggest that larger confirmatory analyses regarding the impacts of employment status on HRQoL using HSUs be conducted with other preferentially sensitive MAUIs.

### Strengths and limitations

This is the first study to investigate HRQoL for pwMS in NZ using the latest iteration of the EQ-5D (the 5L). Our study’s main strengths include a relatively large sample size and a robust methodology. However, this study is affected by three main limitations. First is the use of the Australian tariff for the EQ-5D-5L. However, in the absence of a NZ tariff for the EQ-5D-5L, we have followed the precedent set by a recent clinical trial that adopted the Australian tariff for the NZ cohort (and other country cohorts). Second, the stratification of disability severity into mild, moderate, and severe only (not investigating people with no symptoms as an additional group). In our study cohort, only seven people recorded no symptoms (EDSS = 0), which was insufficient for independent assessment. Consequently, we chose to include these people in the ‘mild’ disability category. Third, the representativeness of our study cohort. This cohort is reasonably representative of the original (NZMSPS) study sample. However, by design it does not include people recently diagnosed with MS and so may not be representatively of the NZ MS community overall.

## Conclusions

HRQoL for pwMS in NZ was lower than comparable countries, including Australia. People with severe MS in NZ reported a very low mean HSU: less than that observed in some terminal cancer cohorts. The EQ-5D-5L had a reduced ceiling effect in this cohort compared to that reported in the broader literature (12% compared to 30%). We suggest a larger comparative study with a preferentially sensitive instrument [42].

## Data Availability

De-identified data is held in a data repository and is available at reasonable request to the Corresponding Author.
